# *Mycobacterium tuberculosis* Complex Lineage 3 as Causative Agent of Pulmonary Tuberculosis, Eastern Sudan^1^

**DOI:** 10.3201/eid2603.191145

**Published:** 2020-03

**Authors:** Yassir A. Shuaib, Eltahir A.G. Khalil, Lothar H. Wieler, Ulrich E. Schaible, Mohammed A. Bakheit, Saad E. Mohamed-Noor, Mohamed A. Abdalla, Glennah Kerubo, Sönke Andres, Doris Hillemann, Elvira Richter, Katharina Kranzer, Stefan Niemann, Matthias Merker

**Affiliations:** Freie Universität Berlin, Berlin, Germany (Y.A. Shuaib, L.H. Wieler);; Research Center Borstel, Borstel, Germany (Y.A. Shuaib, U.E. Schaible, S. Andres, D. Hillemann, S. Niemann, M. Merker);; Sudan University of Science and Technology, Khartoum, Sudan (Y.A. Shuaib, S.E. Mohamed-Noor, M.A. Abdalla);; University of Khartoum, Khartoum, Sudan (E.A.G. Khalil, M.A. Bakheit);; Robert Koch Institute, Berlin (L.H. Wieler);; Kenyatta University, Nairobi, Kenya (G. Kerubo);; Labor Limbach, Heidelberg, Germany (E. Richter);; London School of Hygiene and Tropical Medicine, London, UK (K. Kranzer);; German Center for Infection Research, Borstel Site, Borstel (S. Niemann, M. Merker)

**Keywords:** Mycobacterium tuberculosis complex, lineage 3, transmission, MDR, whole-genome sequencing, Sudan, tuberculosis and other mycobacteria, bacteria, antimicrobial resistance, MDR TB

## Abstract

Pathogen-based factors associated with tuberculosis (TB) in eastern Sudan are not well defined. We investigated genetic diversity, drug resistance, and possible transmission clusters of *Mycobacterium tuberculosis* complex (MTBC) strains by using a genomic epidemiology approach. We collected 383 sputum specimens at 3 hospitals in 2014 and 2016 from patients with symptoms suggestive of TB; of these, 171 grew MTBC strains. Whole-genome sequencing could be performed on 166 MTBC strains; phylogenetic classification revealed that most (73.4%; n = 122) belonged to lineage 3 (L3). Genome-based cluster analysis showed that 76 strains (45.9%) were grouped into 29 molecular clusters, comprising 2–8 strains/patients. Of the strains investigated, 9.0% (15/166) were multidrug resistant (MDR); 10 MDR MTBC strains were linked to 1 large MDR transmission network. Our findings indicate that L3 strains are the main causative agent of TB in eastern Sudan; MDR TB is caused mainly by transmission of MDR L3 strains.

Tuberculosis (TB) remains a major global health problem; 10 million new cases were reported in 2018 (*1*). In Sudan, the estimated national TB incidence in 2018 was 71/100,000 persons; a total of 20,638 cases were reported (*1*). However, the TB burden is by no means homogeneous across the country. For instance, in eastern Sudan, TB notifications reached 275/100,000 persons in 2012 (*2*,*3*). Prevalence of multidrug-resistant TB (MDR TB) (i.e., resistant to isoniazid and rifampin) was estimated at 2.9% in new and 13% in retreatment cases; however, studies have reported MDR TB rates of 6%–22% (*1*,*4**–**10*).

Ongoing transmission is one of the key challenges for TB control programs, especially in countries with a high TB burden (*1*,*11*). In recent years, molecular techniques have been increasingly used to clarify and trace transmission of *Mycobacterium tuberculosis* complex (MTBC) strains and to direct and guide targeted TB control actions (*12*,*13*). However, availability of molecular techniques is limited in many countries in Africa with a high TB burden (*11*).

In Sudan, drug-resistant TB often goes undetected, resulting in inadequate treatment, illness, death, and ongoing transmission (*1*,*14*). Local laboratories have limited access to mycobacterial culture and drug susceptibility testing (DST) or DNA-based techniques (*14*). Therefore, MDR TB rates might be underestimated in eastern Sudan. In addition, mutations that mediate drug resistance have not been investigated.

Taken together, these factors indicate that, although TB is a huge health problem in eastern Sudan, precise data on the phylogeny and transmission dynamics of MTBC strains, as well as on resistance patterns, is sparsely available (*2*,*3*,*7*,*8*,*15*). Studies using molecular epidemiologic tools are rare and have used classical genotyping techniques, such as spoligotyping, which cannot deduce direct transmission events (*5*,*15*). New techniques, such as whole-genome sequencing (WGS), offer the highest resolution for MTBC genotyping and provide precise information on resistance mutations (*16*,*17*). We applied state-of-the-art phenotypic and molecular assays to investigate specimens collected from patients with symptoms suggestive of pulmonary TB, including new and retreatment cases, to analyze the MTBC population structure, putative transmission events, and DST profiles in eastern Sudan.

## Methods

### Study Design and Setting

We recruited patients with symptoms suggestive of pulmonary TB who had positive sputum smears and agreed to participate in this cross-sectional study. Patients had been treated in the outpatient departments at public hospitals in Kassala, Port Sudan, and El-Gadarif in eastern Sudan over 2 recruitment periods, June–October 2014 and January–July 2016. We collected spot and early morning sputum samples. If 1 sample was smear positive, the 2 samples were pooled and stored for <6 months at −20°C. Shortly before we shipped each sample to the National Reference Center (NRC) for Mycobacteria, Borstel, Germany, we transferred a volume of <2 mL to a screw-capped Eppendorf tube; the samples were shipped in 2 separate batches.

### Mycobacterial Culture and Identification

Sample decontamination, smear microscopy, and mycobacterial culture were performed at the NRC (*18*,*19*). We extracted DNA using a QIAamp DNA Mini Kit 250 (QIAGEN, https://www.qiagen.com) according to the instructions of the manufacturer for quantitative PCR (qPCR). We extracted DNA by the boiling/sonication method for conducting line probe assays (LPAs) such as GenoType *Mycobacterium* CM and GenoType *Mycobacterium* MTBC (Hain Lifescience, https://www.hain-lifescience.de) (*19*). We used cetyl trimethylammonium bromide for DNA extraction for WGS (*20*). We transferred the extracted DNA to new Eppendorf tubes and stored it at −20°C until used.

We used an in-house qPCR detecting MTBC and nontuberculous mycobacteria (NTM) DNA to test available culture negative/contaminated samples (*21*). We ran the qPCR experiments using the Rotor-Gene 2000 (Corbett Research Pty Ltd, http://www.australianexporters.net). We used LPAs (Hain Lifescience, https://www.hain-lifescience.de) according to the manufacturer’s instructions to classify isolated mycobacteria into MTBC or NTM and to differentiate the MTBC species. 

We identified NTM species using 16S rRNA, internal transcribed spacer (ITS) DNA fragment sequencing, or both (*22*). We sequenced the complete PCR products on an automated DNA sequencer (ABI 377; Applied Biosystems, https://www.thermofisher.com) by cycle sequencing using the Big Dye RR Terminator Cycle Sequencing Kit (Applied Biosystems). We aligned the resulting sequences and compared them with the sequences of the International Nucleotide Sequence Database Collaboration.

### Drug Susceptibility Testing

We performed phenotypic DST (pDST) for resistance to streptomycin 1 μg/mL, isoniazid 0.1 μg/mL, rifampin 1 μg/mL, ethambutol 5 μg/mL, and pyrazinamide 100 μg/mL for all MTBC isolates (*18*). We further investigated isolates with resistance to >1 first-line drug for resistance to ofloxacin 2 μg/mL, amikacin 1 μg/mL, and capreomycin 2.5 μg/mL (*23*).

We further evaluated strains that had a mutation in the *embB* codons 306, 406, or 497 (as detected by WGS) but tested phenotypically susceptible to ethambutol at the critical concentration by determining ethambutol MICs. We assessed concentrations of 1.25, 2.5, 3.75, and 5.0 μg/mL in the BACTEC MGIT 960 system (Becton Dickinson, https://www.bd.com*)* (*24*,*25*).

### WGS

We performed WGS using the Illumina Nextera (XT) kit (https://www.illumina.com) (*26*). We sequenced isolates with a minimum average genome coverage of 50×. We used single-nucleotide polymorphisms (SNPs) occurring in >4 forward and >4 reverse reads, 4 reads calling the allele with a Phred score >20, and a minimum variant frequency of 75% for a concatenated sequence alignment (*27*). In the comparative genomic analysis, we allowed 5% of all samples to miss these coverage and frequency thresholds at individual positions and called the majority allele (>50% variant frequency) to not lose sequence information in genome regions with lower average coverage. We excluded repetitive region and drug resistance associated genes for phylogenetic reconstruction.

### Phylogenetic Inference

We calculated a maximum-likelihood tree with FastTree using the concatenated sequence alignment and a general time-reversible substitution model (*28*). We conducted inspection and rooting of the maximum likelihood tree using FigTree software and performed the graphical presentation using the online tool EvolView (*29*). We calculated maximum parsimony trees with BioNumerics version 7.6 (Applied Maths, https://www.applied-maths.com) using the concatenated sequence alignment (*30*).

### Molecular Drug Resistance Prediction

We screened the *rpsL*, *rrs*, and *gidB* genes for mutations that confer resistance to streptomycin and the *katG* and *inhA* genes and the *fabG*1-*inhA* promoter for resistance to isoniazid (*31*). We inferred rifampin resistance by mutations in the *rpoB* gene. Moreover, we also noted putative compensatory mutations in the *rpoA* and *rpoC* genes (for rifampin resistance) and the *ahpC* gene (for isoniazid resistance). We investigated the *embA*, *embB*, and *embC* genes for resistance conferring mutations to ethambutol and screened the *pncA* gene for mutations associated with resistance to pyrazinamide (*31*). We investigated the *gyrA* and *gyrB* genes for resistance to fluoroquinolones and investigated the *rrs* gene for resistances against kanamycin, amikacin, and capreomycin. In addition, we screened the *eis* promoter region for resistance against KAN and the *tlyA* for resistance against capreomycin. For ethionamide, we investigated the *ethA* and *inhA* genes and the *fabG*1-*inhA* promoter and for para-aminosalicylic acid, we investigated the *ribD*, *thyA*, *thyX*, and *folC* genes (*31*).

### Statistics

We used SPSS version 20.0 (https://www.ibm.com) for all appropriate statistical analyses. We obtained descriptive statistics of the variables, including frequencies and proportions. We analyzed differences between groups by using the χ^2^ or Fisher exact test; p<0.05 denoted statistical significance (*32*).

### Ethics Considerations

Scientific and ethics approval for the study was provided by the National Research Ethics Committee, Federal Ministry of Health, Khartoum, Sudan, and by the Institutional Review Board of the Institute of Endemic Diseases, University of Khartoum, Khartoum, Sudan (no. 85–03–09). We obtained written informed consent for participation in the study from participants or, in case of children or illiterate patients, their guardians.

## Results

### Study Population

**Figure 1:**
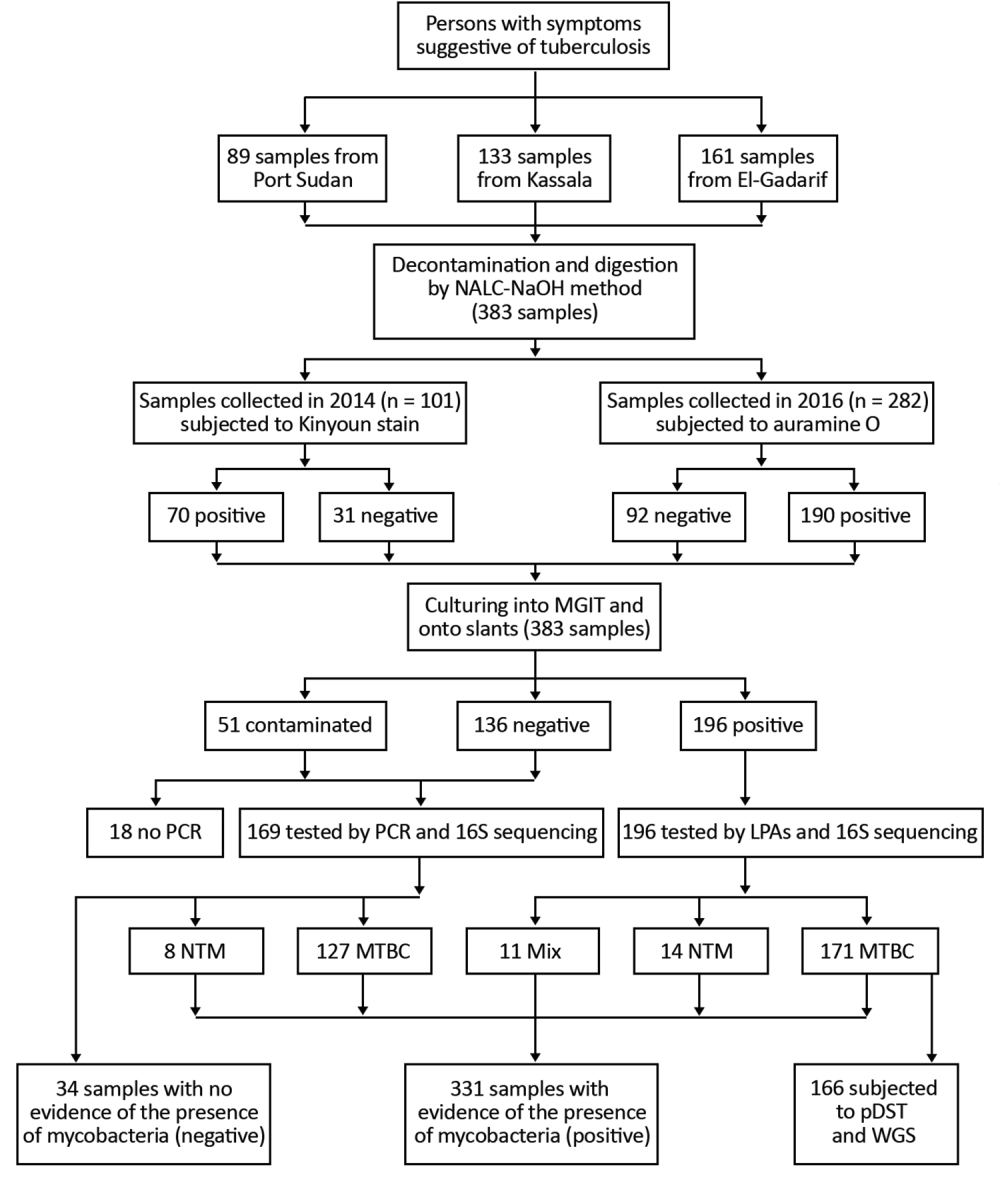
Work flow for study of *Mycobacterium tuberculosis* complex lineage 3 as causative agent of pulmonary tuberculosis, eastern Sudan. LPAs, HAIN line probe assay for GenoType CM and GenoType MTBC; MGIT, mycobacteria growth indicator tube; MTBC, *Mycobacterium tuberculosis* complex; mix, 2 different bacteria grew on the same culture; NALC-NaOH, sodium hydroxide/N-acetyl-cysteine; NTM, nontuberculous mycobacteria; pDST, phenotypic drug susceptibility testing; WGS, whole genome sequencing. Adopted from Shuaib et al. (*14*).

Sputum samples were provided by smear-positive patients with TB from 3 areas in eastern Sudan in 2014 (n = 101) and 2016 (n = 282) (Figure 1). Based on hospital records, we included 10%–20% of all patients who received diagnoses of TB during the study period. We collected patient-derived samples from 161 patients (42%) in El-Gadarif, 133 patients (34.7%) in Kassala, and 89 patients (23.3%) in Port Sudan hospitals. Patients who provided samples had a median age of 35 years (interquartile range 25–45 years); most (245/383; 66%) were male. In addition, 81.5% (312/383) were new and 5.5% (21/383) were retreatment TB cases; data on TB treatment history were unavailable for 13.0% (50/383) (Table 1). Comparison of the 2 patient cohorts revealed no significant difference between the proportions of L3 strains (p = 0.068 by Fisher exact test) but the 2014 cohort contained more drug-resistant (p = 0.019 by Fisher exact test) and clustered (p = 0.016 by Fisher exact test) strains (Table 2).

**Table 1:** Demographic characteristics of tuberculosis patients investigated in eastern Sudan

Characteristic	No. recruited patients	% Recruited patients
Origin		
Kassala	133	34.7
El-Gadarif	161	42.0
Port Sudan	89	23.3
Sex		
M	245	64.0
F	123	32.1
Not available	15	3.9
Age, y		
<25	76	19.8
25–40	170	44.4
>40	108	28.2
Not available	29	7.6
Treatment history		
Retreatment	21	5.5
New	312	81.5
Not available	50	13.0
All patients	383	100

**Table 2:** Comparison of *Mycobacterium tuberculosis* complex isolates sampled in eastern Sudan, 2014 and 2016*

Variable	Year of sample collection, no. (%) isolates		Total no.	p value
	2014	2016		
Lineage				0.068
L3	51 (82.3)	71 (68.3)	122	
Non-L3	11 (17.7)	33 (31.7)	44	
DST				0.019
Any drug resistance	20 (32.3)	16 (15.4)	36	
No drug resistance	42 (67.7)	88 (84.6)	120	
<12 SNP cluster				0.016
Clustered	36 (58.1)	40 (38.5)	76	
Not clustered	26 (41.9)	64 (61.5)	90	
All patients	62 (100)	104 (100)	166	

*DST, drug susceptibility testing; L, lineage; SNP, single-nucleotide polymorphism.

### *Mycobacterium* Isolation and Species Identification

Of all collected specimens, 51.2% (196/383) were culture positive for mycobacteria; LPAs identified most (n = 171, 87.2%) as MTBC (Figure 1). The rest of the specimens were either culture negative or contaminated; we tested them with qPCR and Sanger sequencing for mycobacterial DNA detection and species identification (Figure 1) (*14*).

### MTBC Population Structure and Genome-Based Clusters

**Figure 2:**
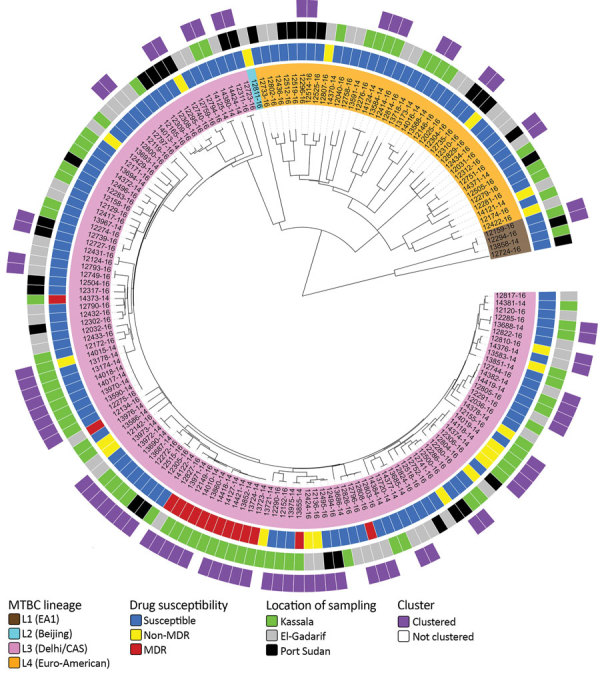
MTBC population structure in eastern Sudan. Maximum-likelihood tree based on 11,932 concatenated single-nucleotide polymorphisms (SNPs) using a general time-reversible substitution model. Colored bars code for (inner to outer ring) MTBC lineages (L1–4); genotypic DST results stratified to MDR, non-MDR, and pansusceptible; sampling location; and clustered and nonclustered strains (SNP distance ≤12). MDR, multidrug resistant; MTBC, *Mycobacterium tuberculosis* complex.

We performed WGS successfully on 166 MTBC strains. We built a maximum-likelihood phylogeny upon a concatenated sequence alignment comprising 11,932 SNPs to investigate the MTBC population structure (Figure 2). We performed MTBC (sub)lineage (L) classification with an SNP bar code nomenclature that was recently introduced (*33*). Strains of L3 (Delhi/CAS) were predominant (73.5%, 122/166), followed by L4 (Euro-American) strains (23.5%, 39/166). We further classified L4 strains into several sublineages (Appendix 1 Figure 1). The remaining isolates belonged to L1 (2.4%, 4/166) and L2 (0.6%, 1/166). 

To obtain an indication about putative recent transmission events, we conducted a cluster analysis based on a pairwise SNP distance of <12 SNPs between any 2 strains (*12*,*34*). Overall, 45.9% (76/166) of the strains were grouped in 29 clusters comprising 2–8 isolates/patients (Appendix 1 Figure 2). L3 strains were observed with a higher clustering rate (52.5%, 64/122) than L4 isolates (28.2%, 11/39) (p = 0.016 by Fisher exact test). Two of the 4 L1 strains were clustered; no L2 strains were assigned to a WGS cluster. Moreover, considering a stricter threshold (<5 SNPs), 36.7% (61/166) isolates were still connected in 24 clusters comprising 2–8 isolates/patients (Appendix 1 Figure 3).

### pDST and Genotypic DST

To determine resistance levels and related genomic variants, we performed pDST and genomic resistance predictions or genotypic DST (gDST) and compiled detailed data on resistances and resistance conferring mutations (Tables 3, 4; Appendix 1 Table). Overall, 21.7% (36/166) of the strains showed resistance to >1 of the tested first-line antimicrobial drugs by pDST, including 15 (9.0%) MDR and 21 (12.7%) non-MDR strains (Appendix 2). Strikingly, all MDR and 76.1% of non-MDR strains belonged to L3. Furthermore, beyond the MDR classification, we found that L3 strains in eastern Sudan were more often found with drug resistances as compared with L4 strains (L3, 31/122, 25.4%; L4, 4/39, 10.3%; p = 0.048 by Fisher exact test). 

**Table 3:** Mutations conferring drug resistance among *Mycobacterium tuberculosis* complex genotypes identified in eastern Sudan, 2014 and 2016*

Genotype	No. (%) strains	No. (%) strains with mutations for drug					No. (%) mutations/genotype	No. (%) strains/genotype	
		SM	INH	RIF	EMB	PZA		Non-MDR	MDR
Delhi/CAS	122 (73.5)	28 (85.0)	17 (100)	17 (100)	12 (100)	1 (100)	75 (93.75)	16 (76.1)	15 (100)
EAI	4 (2.4)	0	0	0	0	0	0	0	0
LAM	5 (3.0)	0	0	0	0	0	0	0	0
Uganda	4 (2.4)	1 (3.0)	0	0	0	0	1 (1.25)	1 (4.8)	0
S-type	2 (1.2)	0	0	0	0	0	0	0	0
Haarlem	5 (3.0)	0	0	0	0	0	0	0	0
Sudan H37Rv-like	4 (2.4)	0	0	0	0	0	0	0	0
X-type	2 (1.2)	1 (3.0)	0	0	0	0	1 (1.25)	1 (4.8)	0
Cameroon	2 (1.2)	0	0	0	0	0	0	0	0
Euro-American	15 (9.0)	2 (6.0)	0	0	0	0	2 (2.5)	2 (9.5)	0
Beijing	1 (0.6)	1 (3.0)	0	0	0	0	1 (1.25)	1 (4.8)	0
Total	166 (100)	33 (100)	17 (100)	17 (100)	12 (100)	1 (100)	80 (100)	21 (100)	15 (100)

*CAS, Central Asian strain; EAI, East African Indian; EMB, ethambutol; INH, isoniazid; LAM, Latin American Mediterranean; MDR, multidrug-resistant; PZA, pyrazinamide; RIF, rifampin; SM, streptomycin.

**Table 4:** Performance of genotypic drug resistance prediction to first-line tuberculosis drugs in *Mycobacterium tuberculosis* complex strains, eastern Sudan*

Drug	Resistant				Susceptible			Se, %† (95% CI)	Sp, %‡ (95% CI)	PPV, %§ (95% CI)	NPV, %¶ (95% CI)	Unknown mutations (%)
	gR (TP)	gS (FN)	gU (FN)		gR (FP)	gS (TN)	gU (TN)					
SM	24	0	9		0	133	0	72.7 (54.5–86.7)	100	100	93.7 (89.4–96.3)	9/166 (5.4)
INH	17	0	0		0	149	0	100	100	100	100	0/166 (0.0)
RIF	17	0	0		0	149	0	100	100	100	100	0/166 (0.0)
EMB	1	0	0		10*	155	0	100	93.9 (89.1–97.1)	9.10 (5.2–15.4)	100	0/166 (0.0)
PZA	1	0	0		0	165	0	100	100	100	100	0/166 (0.0)

*See MICs in the Methods section. EMB, ethambutol; FN, false negative; FP, false positive; gR, genetically resistant; gS, genetically susceptible; gU, genetic resistance unknown; INH, isoniazid; NPV, negative predictive value; PPV, positive predictive value; PZA, pyrazinamide; RIF, rifampin; Se, sensitivity; Sp, specificity; SM, streptomycin; TN, true negative; TP, true positive.  †Se = TP ÷ TP + FN.  ‡Sp = TN ÷ TN + FP. §PPV =  TP ÷ (TP + FP). ¶NPV =  TN ÷ (TN + FN).

We detected resistance to streptomycin in 19.9% (33/166) of the strains, mediated by mutations in *rspL* (Lys43Arg, Lys88Arg, and Lys88Met), *gidB* (e.g., Ala138Val), and *rrs* (514, a/c) genes. We observed all isoniazid-resistant strains (10.2%, 17/166) either with a mutation in *katG* (Ser315Thr and Ser315Asn) that changes catalase–peroxidase activities or in the promoter region of the drug target InhA, *fabG1*-*inhA* (−15 c/t), which also confers resistance to the second-line drug ETH. Resistance to rifampin was found in 10.2% (17/166) of the strains and was mediated by mutations in the *rpoB* gene (Ser450Leu, His445Tyr, His445Asn, and His445Asp). We found 1 ethambutol-resistant strain (0.6%) with the mutation *embB* Gln497Arg. However, we also detected 11 additional mutations associated with ethambutol resistance in the *embB* gene (10 Met306Ile and 1 Met306Val) but with MICs ranging from 1.25 to 5 μg/mL, classifying these strains as phenotypically susceptible based on the recommended critical concentration for ethambutol. With regard to pyrazinamide, we identified 1 strain (0.6%) with the mutation *pncA* Gln10Arg, coinciding with phenotypic pyrazinamide resistance.

A detailed comparison of the pDST and gDST results revealed a high sensitivity and specificity for isoniazid, rifampin, and pyrazinamide resistance prediction by WGS (Table 4). For ethambutol, we determined high-confidence resistance SNPs at codon 306, 406, or 497; however, varying levels of ethambutol MICs in the strains with mutations resulted in a very low positive predictive value. For streptomycin, we considered the *gidB* mutations (Phe12Ser, Arg39Pro, Trp45STP, Ser136STP, Iso114Ser, and deletions at positions 4408101, 4408017, and 4408116) to be mutations with an unclear effect. However, these strains eventually tested phenotypically resistant to streptomycin, leading to a reduced sensitivity.

All strains with resistances to >1 first-line antimicrobial drug were phenotypically and genotypically susceptible to ofloxacin, capreomycin, and amikacin. We identified no genotypic resistance marker mediating para-aminosalicylic acid resistance.

Based on a 12-bp SNP threshold between any 2 strains, 80.0% (12/15) of the MDR strains were clustered or connected (i.e., associated with recent transmission); based on <5 SNPs distance, 60% (9/15) of the MDR strains were clustered (Appendix 1 Figure 4, panel A). Most of the clustered strains at <12 SNPs were isolated from patients in Kassala and grouped in clusters 4 and 29. These strains also shared the same *rpsL* (Lys43Arg) and the *katG* (Ser315Thr) mutations but harbored different mutations in the *rpoB* gene; strains of cluster 4 had the Ser450Leu mutation, whereas strains of cluster 29 exhibited a His445Tyr mutation. This finding points toward a close relationship between the strains of both clusters that likely emerged from a common recent ancestor already being polyresistant to streptomycin and isoniazid (Appendix 1 Figure 4, panel B). Furthermore, all 5 strains of cluster 29 had the *embB* Met306Ile mutation, but 1 of them also had the mutation *embB* Gly406Asp. Within cluster 4, two strains acquired the mutation *embB* Met306Ile independently, and 1 acquired *embB* Met306Val, as judged by the tree topology (Appendix 1 Figure 4, panel B). Moreover, among all drug-resistant strains, only 1 strain was identified with resistance to pyrazinamide mediated by the mutation *pncA* Gln10Arg.

## Discussion

By using conventional diagnostics and WGS, we showed that pulmonary TB in eastern Sudan is caused predominantly by L3 strains (Delhi/CAS). Drug resistance and recent transmission were associated with L3 strains, accentuating the key role of L3 strains in TB epidemiology in eastern Sudan. In addition, most MDR TB cases were connected in 2 closely related molecular clusters (denoting recent transmission of MDR strains). This finding suggests that more focused infection control measures and contact tracing of patients with MDR TB need to be introduced to break the transmission chains at an early stage.

In this study, we found that a high proportion of culture-positive pulmonary TB cases (73.4%) were caused by L3 strains. This finding is in line with previous studies that have been based on classical genotyping methods and reported rates of 40% or higher of the so-called Central Asia spoligotype family in central and eastern Sudan (*5*,*15*,*35*). Moreover, Couvin et al. (*36*) identified Sudan as an L3 hotspot in Africa. In this regard, it is intriguing to speculate whether L3 strains in general or certain subgroups have developed particular pathobiologic properties rendering them more virulent in East Africa host populations. Recently, Stucki et al. (*37*) hypothesized a concept of generalists and specialist among L4 strains based on the width of their geographic distribution. For L3 strains, larger studies are needed to reveal their global genetic diversity and geographic prevalence, which might inform about particular successful L3 subgroups and related causative variants in their genomes.

In addition to the general dominance of L3 strains in eastern Sudan, we found that all MDR TB cases were caused exclusively by L3 strains. Of major concern is that 10 of 15 MDR strains were part of 2 genetically related clusters isolated mainly from patients treated in 2014 in Kassala hospital. At first glance, this finding suggested nosocomial transmission. However, 2 strains of these clusters were isolated in 2016, including a strain from a patient treated in El-Gadarif hospital who had also acquired resistance to ethambutol and pyrazinamide. This patient was the first patient in our study cohort infected with a fully first-line drug-resistant strain, clearly emphasizing the importance of adopting focused TB control measures, including rapid detection and effective treatment of patients with MDR TB, to better contain transmission of MDR strains and prevent development of further drug resistances in the region. However, these measures are far from reality because proper TB diagnostics are virtually absent in eastern Sudan; other impeding factors are social stigma, lack of motivation, and poor awareness of TB treatment, with default rates of 14%–57% (*38*, *39*). This situation may even lead to a further aggravation of the drug resistance problem through selection of MDR clones with additional drug resistances in failing treatment regimens and further transmission of fully first-line resistant MDR strains (*14*,*39*). However, our WGS analysis revealed that MDR isolates did not exhibit mutations mediating resistances to second-line drugs (except for isoniazid/ETH cross resistance), leaving reasonable therapeutic options for patients in eastern Sudan with MDR TB.

Former studies in central and eastern Sudan reported drug resistances in 39%–67% and MDR in 6%–22% of the strains investigated (*4**–**10*). Those variations could possibly be attributed to dissimilarities in study design, sample size, and characteristics of study populations. In former studies, 53–235 samples from only new or new and retreatment TB cases with unknown HIV status or with a proportion of HIV-positive cases were investigated (*4**–**10*). Additionally, variations could also be linked to the laboratory technique used for pDST; for example, discordant results have been noticed between the BACTEC MGIT 960 and the proportion methods for streptomycin and ethambutol (*40*). In Sudan, only 1 study used the BACTEC MGIT 960; the remaining studies used the proportion method on Löwenstein–Jensen slants (*4**–**10*).

Considering the lack of pDST and the technical challenges associated with its implementation in Sudan, introduction of rapid molecular diagnostics to find patients with MDR TB is crucial for timely detection, treatment, and control. Moreover, rapid diagnostics will ultimately strengthen the national TB control program in Sudan. In line with previous studies, our data demonstrate an excellent performance of gDST for molecular resistance prediction (*16*,*17*,*41*). One example of the benefits of molecular assays is the correction of false ethambutol susceptibility results based on pDST in strains that harbor high-confidence *embB* resistance (*42*). Previous studies already revealed a low performance of ethambutol pDST, attributable mainly to the small difference between the wild-type and mutant MIC levels, leading to the effect that strains with canonical *embB* mutations show ethambutol MICs around the defined breakpoint of 5.0 μg/mL, resulting in a low reproducibility of phenotypic results (*24*,*25*,*42*). Therefore, classical Sanger sequencing of the *embB* codons 306, 354, and 406 was recently proposed to overrule phenotypic ethambutol susceptibility results in cases of presence of mutations in these codons (*42*). Furthermore, Cepheid GeneXpert and Hain MTBDR*plus* version 2.0 would have recognized all rifampin-resistant mediating mutations in our study setting and, therefore, offer a rapid solution for identification of patients with MDR/rifampin-resistant TB in eastern Sudan.

This multisite study was conducted in 3 public hospitals in eastern Sudan, comprising 10%–20% of the TB cases in the region during the study period; it thus represents a snapshot of the population diversity and transmission dynamics of MTBC strains in eastern Sudan. An additional strength of this study is that cultures, DSTs, and WGS were done in a World Health Organization–certified NRC in a high-resource setting in Germany, enabling maximum resolution for characterization of MTBC strains.

This study had >2 limitations. First, the prolonged transit time of patient-derived samples from Sudan to the NRC in Germany affected the viability of the MTBC bacteria; therefore, no mycobacterial growth was detected for some samples. Furthermore, the unavailability of clinical data, such as HIV status and treatment outcomes, prohibited further linking of bacteriological results to these clinical data.

In conclusion, L3 strains play a pivotal role in the epidemiology and transmission of TB, particularly MDR TB, in eastern Sudan. Transmission of MDR TB could possibly be an emerging concern for local TB departments and hospitals. Therefore, to contain MDR TB transmission, rapid molecular diagnostics, such as Cepheid GeneXpert or Hain MTBDR*plus* v2.0, are desirable in combination with focused tracing of contacts of patients with MDR TB. In addition, early onset of MDR TB therapy would be an ideal approach to reduce the number of secondary cases.

Appendix 1 Detected mutations and phylogenetic trees showing various aspects of *Mycobacterium tuberculosis* lineages in study of *Mycobacterium tuberculosis* complex lineage 3 as causative agent of pulmonary tuberculosis, eastern Sudan.

Appendix 2 Phylogenetic classification, phenotypic drug susceptibility test results, and molecular drug resistance markers in study of *Mycobacterium tuberculosis* complex lineage 3 as causative agent of pulmonary tuberculosis, eastern Sudan.
