# Supplementary material for: Mycobacterium tuberculosis Complex Lineage 3 as Causative Agent of Pulmonary Tuberculosis, Eastern Sudan
Source: Emerg Infect Dis. 2020 Mar;26(3):427–36. doi: 10.3201/eid2603.191145 (PMC7045825; doi:10.3201/eid2603.191145)
Supplement: Appendix 1 — Detected mutations and phylogenetic trees showing various aspects of Mycobacterium tuberculosis lineages in study of Mycobacterium tuberculosis complex lineage 3 as causative agent of pulmonary tuberculosis, eastern Sudan. [file 19-1145-Techapp-s1.pdf]

Article DOI: https://doi.org/10.3201/eid2603.191145

Mycobacterium tuberculosis Complex Lineage 3 as Causative Agent of Pulmonary Tuberculosis, Eastern Sudan

Appendix 1

Appendix 1 Table. Detected mutations that mediate resistance to first-line anti-TB drugs in 15 MDR MTBC strains, eastern Sudan*

		SM		INH		RMP		EMB		PZA			
No.	Key	pDST	gDST	pDST	gDST	pDST	gDST	pDST	gDST	pDST	gDST	Coll lineage	≤12 SNP cluster/group number
1	13852/14	R	rpsL Lys43Arg (aag/aGg)	R	katG Ser315Thr (agc/aCc)	R	rpoB His445Tyr (cac/Tac)	R	embB Met306Ile (atg/atA)	S	WT	Delhi/CAS	Cluster 29
2	13723/14	R	rpsL Lys43Arg (aag/aGg)	R	katG Ser315Thr (agc/aCc)	R	rpoB His445Tyr (cac/Tac)	R	embB Met306Ile (ggc/gAc), Gly406Asp (ggc/gAc)	S	WT	Delhi/CAS	Cluster 29
3	14421/14	R	rpsL Lys43Arg (aag/aGg)	R	katG Ser315Thr (agc/aCc)	R	rpoB His445Tyr (cac/Tac)	R	embB Met306Ile (atg/atA)	S	WT	Delhi/CAS	Cluster 29
4	14127/14	R	rpsL Lys43Arg (aag/aGg)	R	katG Ser315Thr (agc/aCc)	R	rpoB His445Tyr (cac/Tac)	R	embB Met306Ile (atg/atA)	S	WT	Delhi/CAS	Cluster 29
5	13724/14	R	rpsL Lys43Arg (aag/aGg)	R	katG Ser315Thr (agc/aCc)	R	rpoB His445Tyr (cac/Tac)	R	embB Met306Ile (atg/atA)	S	WT	Delhi/CAS	Cluster 29
6	12827/16	R	rpsL Lys43Arg (aag/aGg)	R	katG Ser315Thr (agc/aCc)	R	rpoB Ser450Leu (tcg/tTg)	R	embB Met306Val (atg/Gtg)	R	pncA Gln10Arg (cag/cGg)	Delhi/CAS	Cluster 4
7	12149/16	R	rpsL Lys43Arg (aag/aGg)	R	katG Ser315Thr (agc/aCc)	R	rpoB Ser450Leu (tcg/tTg)	R	embB Met306Ile (atg/atA)	S	WT	Delhi/CAS	Cluster 4
8	14010/14	R	rpsL Lys43Arg (aag/aGg)	R	katG Ser315Thr (agc/aCc)	R	rpoB Ser450Leu (tcg/tTg)	S	WT	S	WT	Delhi/CAS	Cluster 4
9	13971/14	R	rpsL Lys43Arg (aag/aGg)	R	katG Ser315Thr (agc/aCc)	R	rpoB Ser450Leu (tcg/tTg)	R	embB Met306Ile (atg/atA)	S	WT	Delhi/CAS	Cluster 4
10	13860/14	R	rpsL Lys43Arg (aag/aGg)	R	katG Ser315Thr (agc/aCc)	R	rpoB Ser450Leu (tcg/tTg)	S	WT	S	WT	Delhi/CAS	Cluster 4

No.	Key	SM		INH		RMP		EMB		PZA		Coll lineage	≤12 SNP cluster/group number
		pDST	gDST	pDST	gDST	pDST	gDST	pDST	gDST	pDST	gDST		
1	12808/16	S	WT	R	katG Ser315Thr (agc/aCc)	R	rpoB His445Asp (cac/Gac)	S	WT	S	WT	Delhi/CAS	Cluster 19
12	13855/14	R	rpsL Lys43Arg (aag/aGg)	R	fabG1 Thr4Iso (aca/aTa)	R	rpoB Ser450Leu (tcg/tTg)	S	WT	S	WT	Delhi/CAS	Cluster 3
13	12134/16	R	gidB Ala138Val (gcg/gTg)	R	katG Ser315Asn (agc/aCc)	R	rpoB His445Asn (cac/Cac)	R	embB Gln497Arg (cag/cGg)	S	WT	Delhi/CAS	Unique
14	14418/14	R	rpsL Lys43Arg (aag/aGg)	R	katG Ser315Thr (agc/aCc)	R	rpoB His445Tyr (cac/Tac)	R	embB Met306Ile (atg/atA)	S	WT	Delhi/CAS	Unique
15	14373/14	S	WT	R	katG Ser315Thr (agc/aCc)	R	rpoB Ser450Leu (tcg/tTg)	S	WT	S	WT	Delhi/CAS	Unique

*EMB, ethambutol; gDST, genotypic drug susceptibility testing; INH, isoniazid; pDST, phenotypic drug susceptibility testing; PZA, pyrazinamide; R, resistant; RMP, rifampin; S, susceptible; SM, streptomycin; SNP, single-nucleotide polymorphism; WT, wild-type.

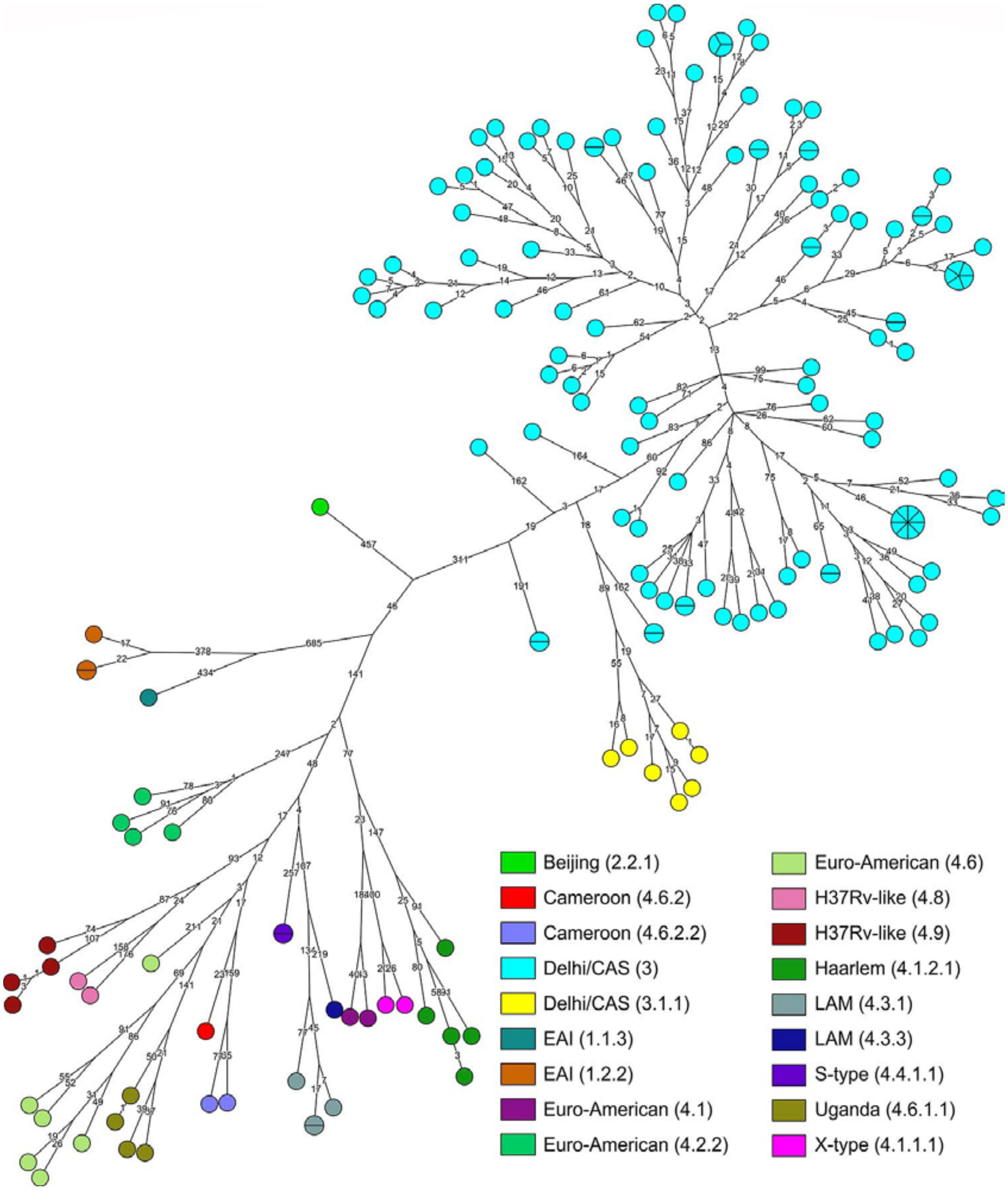

Appendix Figure 1. Phylogenetic tree based on maximum parsimony showing MTBC lineage classification of all analyzed MTBC isolates. A maximum parsimony tree was calculated based on 11,932 concatenated SNPs showing the identified MTBC lineages (L1–4). The genetic distance (SNP differences) is indicated on tree branches. Different lineages and sublineages are indicated in different colors with a SNP bar code. MTBC, *Mycobacterium tuberculosis* complex; SNP, single-nucleotide polymorphism.

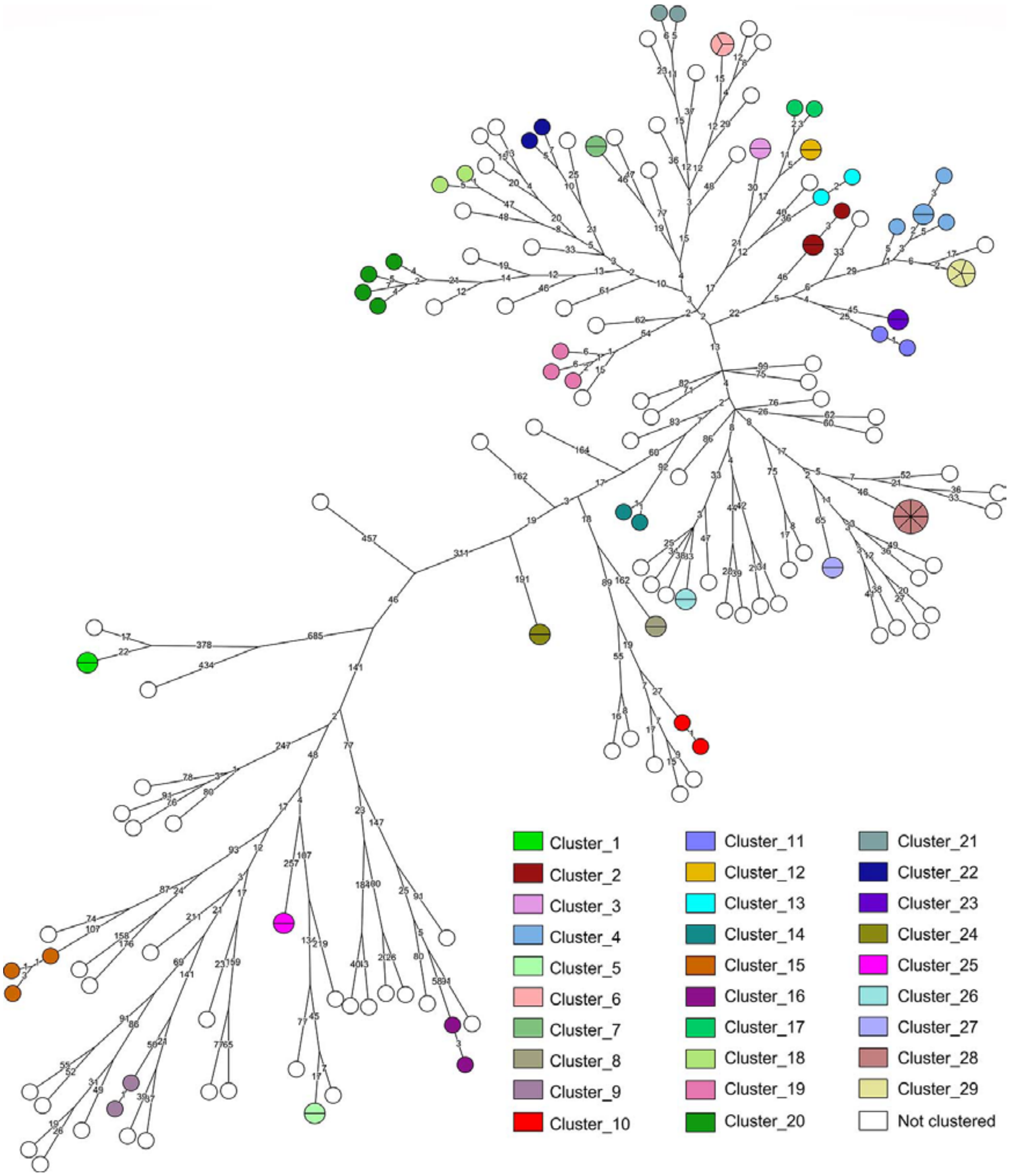

Appendix Figure 2. Phylogenetic tree based on maximum parsimony showing ≤12 SNP clusters. A maximum parsimony tree was calculated based on 11,932 concatenated SNPs showing molecular clusters. The genetic distance (SNP differences) is indicated on tree branches. Different clusters are indicated in different colors. SNP, single-nucleotide polymorphism.

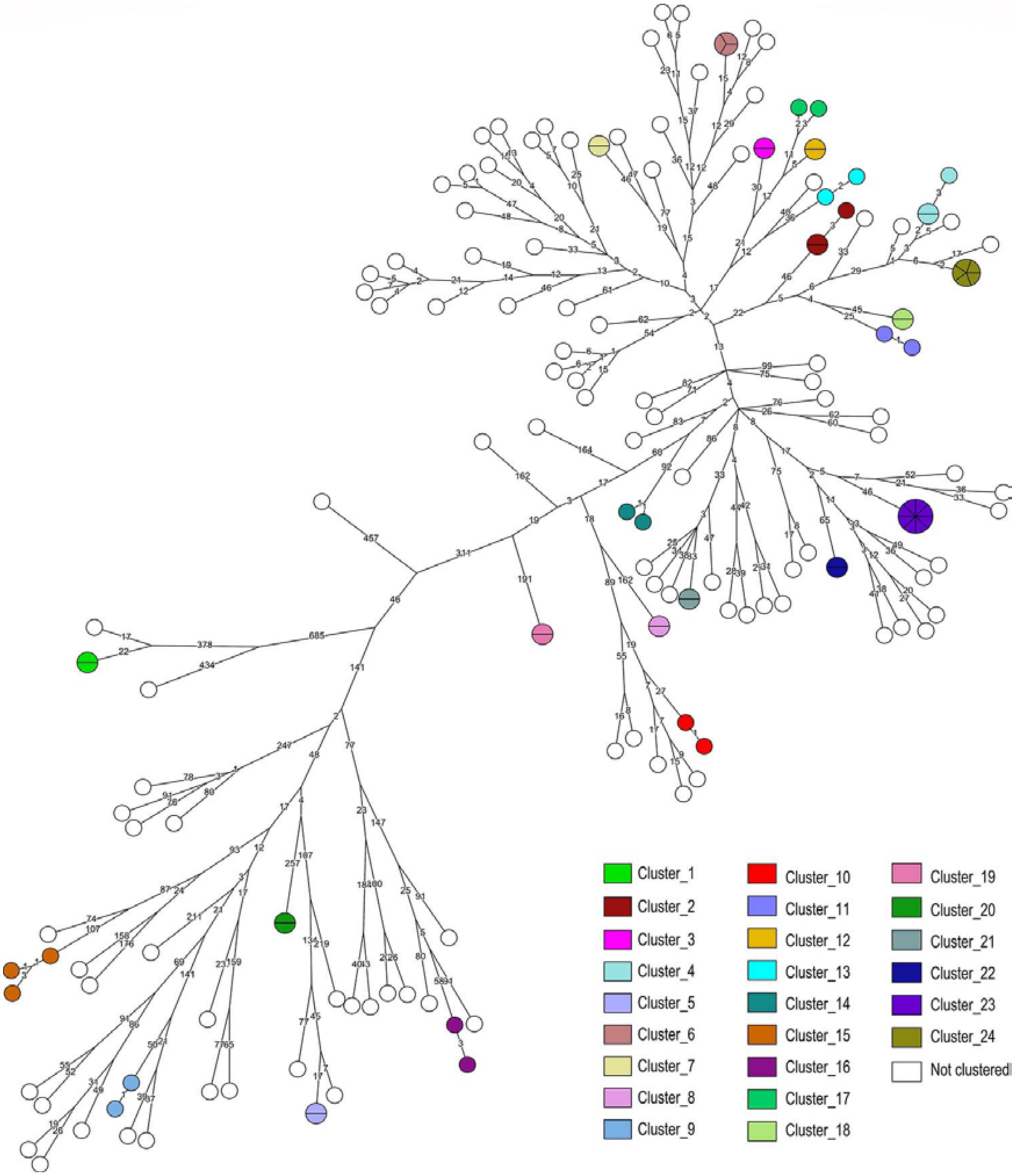

Appendix Figure 3. Phylogenetic tree based on maximum parsimony showing <u><</u>5 SNP clusters. A maximum parsimony tree was calculated based on 11,932 concatenated SNPs showing molecular clusters. The genetic distance (SNP differences) is indicated on tree branches. Different clusters are indicated in different colors. SNP, single-nucleotide polymorphism.

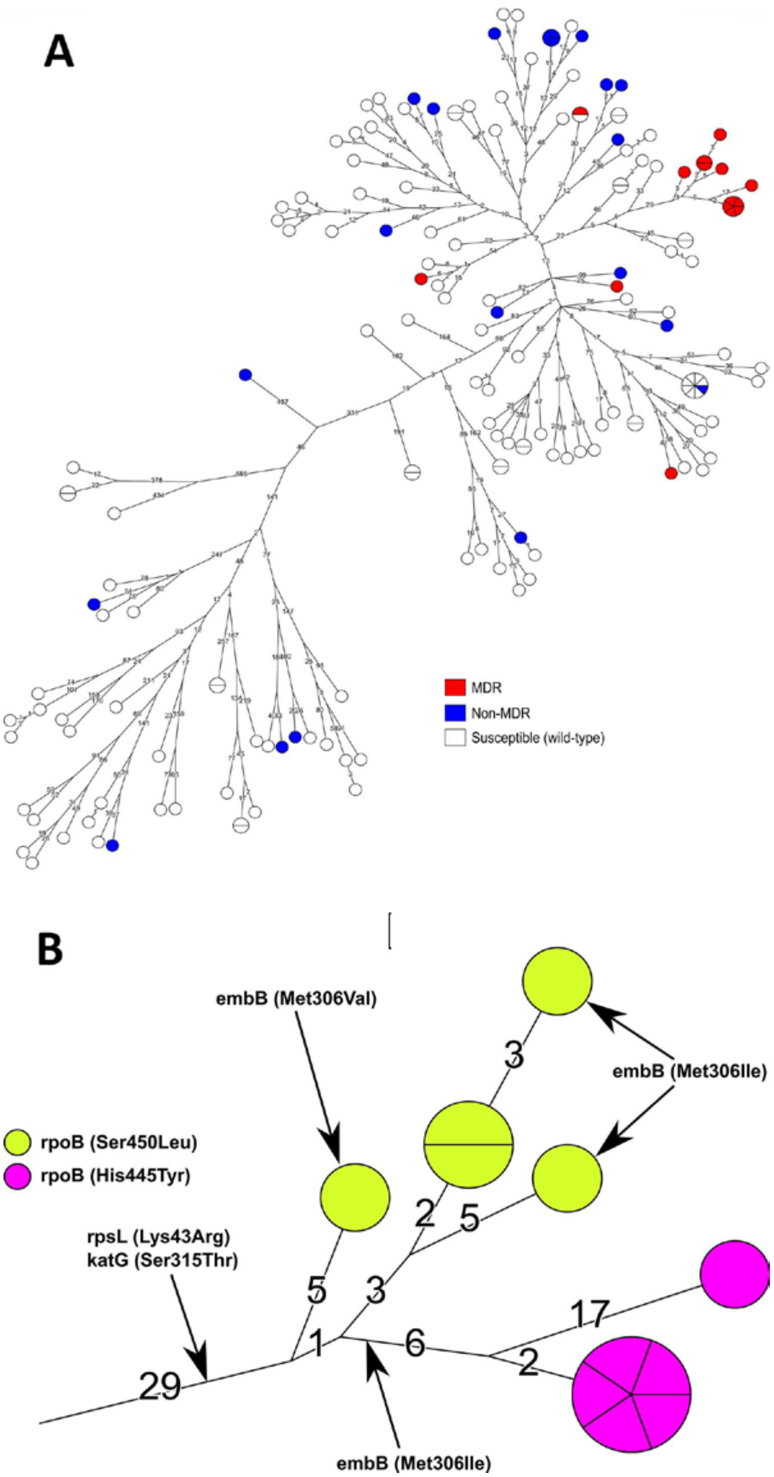

Appendix Figure 4. Phylogenetic tree based on maximum parsimony showing genotypic drug resistances. A) Maximum parsimony tree based on 11,932 concatenated SNPs showing strains that harbor drug resistance mutations (MDR and non-MDR isolates) and wild-type (i.e., isolates with no drug resistance mutations). The genetic distance (SNP differences) is indicated on the branches. B) Strains of 2 MDR clusters emerging from a polyresistant common ancestor that exhibits mutations mediating

resistances to SM and INH. Strains of the 2 clusters are differentiated by distinct *rpoB* mutations mediating RMP resistance. The 5 strains of the pink cluster (cluster 29) share the mutation *embB* Met306Ile but 1 of them also has the Gly406Asp *embB* mutation. Of note, the unusual high genetic distance of 17 SNPs distinguishing 1 pink isolate are caused by low-frequency SNPs. This probably reflects a mixed infection with very closely related strains, as most of the strain population was identical to the strains' populations of isolates in the pink cluster (cluster 29). INH, isoniazid; MDR, multidrug resistance; RMP, rifampin; SM, streptomycin; SNP, single-nucleotide polymorphism.
